# Measuring Catastrophic Costs Due to Tuberculosis in Myanmar

**DOI:** 10.3390/tropicalmed6030130

**Published:** 2021-07-14

**Authors:** Si Thu Aung, Aung Thu, Htin Lin Aung, Min Thu

**Affiliations:** 1 Disease Control, Department of Public Health, Ministry of Health and Sports, Nay Pyi Taw 15013, Myanmar; 2 TB Unit, WHO, Yangon 11201, Myanmar; thua@who.int (A.T.); min.thu@sgs.tu.ac.th (M.T.); 3 Department of Microbiology and Immunology, University of Otago, Dunedin 9016, New Zealand

**Keywords:** tuberculosis (TB), catastrophic cost, TB patient, social protection

## Abstract

Background: This is the first survey to use the World Health Organization (WHO) methodology to document the magnitude and main drivers of tuberculosis (TB) patient costs in order to guide policies on cost mitigation and to produce a baseline measure for the percentage of TB-affected households experiencing catastrophic costs in Myanmar. Methods: A nationally representative cross-sectional survey was administered to 1000 TB patients in health facilities from December 2015 to February 2016, focusing on costs of TB treatment (direct and indirect), household income, and coping strategies. A total cost was estimated for each household by extrapolating reported costs and comparing them to household income. If the proportion of total costs exceeded 20% of the annual household income, a TB-affected household was deemed to have faced catastrophic costs. Results: 60% of TB-affected households faced catastrophic costs in Myanmar. On average, total costs were USD 759, and the largest proportion of this total was accounted for by patient time (USD 365), followed by food costs (USD 200), and medical expenses (USD 130). Low household wealth quintile and undergoing MDR-TB treatment were both significant predictors for households facing catastrophic costs. Conclusions: The high proportion of TB-affected households experiencing catastrophic costs suggests the need for TB-specific social protection programs in patient-centered healthcare. The survey findings have led the government and donors to increase support for MDR-TB patients. The significant proportion of total spending attributable to lost income and food or nutritional supplements suggests that income replacement programs and/or food packages may ameliorate the burdensome costs.

## 1. Introduction

Tuberculosis (TB) kills approximately 1.5 million people per year [1]. Beyond being the greatest infectious disease killer, TB often inflicts significant social and economic costs on affected households. The economic burden of illness due to TB can be devastating. In low- and middle-income countries, TB patients face costs that, on average, amount to half of their annual income [2]. Although in many countries TB diagnostic tests and drug treatment are provided for free, this has been shown to be insufficient in protecting TB-affected households economic burden [3]. One reason for this is that TB costs go beyond direct health expenditure, and include payments for transport, accommodation, and food expenses, as well as income loss from illness and care-seeking. Failure to reduce these economic barriers can mean access to care is inhibited, diagnosis and treatment are delayed or foregone, and TB transmission in the community is exacerbated.

The global prevalence of multidrug-resistant TB (MDR-TB) is of growing concern. MDR-TB is caused by *Mycobacterium tuberculosis* that is resistant to at least isoniazid (INH) and rifampicin (RIF), the cornerstone medicines for the treatment of TB. Myanmar is one of 14 countries with a high burden of TB, MDR-TB, and TB/HIV. According to the Global TB Report 2020, the prevalence of MDR-TB in Myanmar in new cases and in previously treated cases were 4.9% (4.7–5.1, 95% CI) and 18% (17–19, 95% CI) respectively [1]. The challenges associated with MDR-TB for both treatment providers and patients are significant, with the expense of medications used for MDR-TB, the significant human resources requirements for DOTS treatment, and the lengthy treatment duration for patients.

The Myanmar Health System is made up of a mix of public, private for-profit, private not-for-profit and ethnic health organizations (EHO) which are responsible for providing health services. The Ministry of Health and Sports (MoHS) has since 2014 been leading a technical exercise to define an Essential Package of Health Services (EPHS). The plan is to have a Basic EPHS by 2020, an Intermediate EPHS by 2025 and a Comprehensive EPHS by 2030. The current public sector health service provision focuses on tertiary care, which means that station hospitals and below have received less attention over the past few decades. This underinvestment has led to shortcomings in service availability, readiness, and coverage. Furthermore, there is limited public sector service delivery in both conflict-affected and post-conflict-affected areas. It is recognized that the public sector will not be able to reach the entire population with the Basic EPHS by itself. Public sector facilities vary in terms of their level of readiness. Other actors, such as private for-profit providers, non-government organizations (NGOs), and EHOs are also involved in service delivery, but government oversight and engagement are limited. Among all types of service providers, quality of care shows great variation. EHOs have long been providing essential services and interventions to populations in conflict-affected areas which public sector services do not reach. Despite recent promising initiatives, standardization of these services among the different EHOs, and between EHOs and public sector services, has faced many political and technical challenges. The different cadres of health workers employed by EHOs are currently trained through parallel systems with limited or no recognition from MoHS. Service provision by EHOs relies heavily on donor support, putting their sustainability at risk.

In 2014 the World Health Assembly approved the End TB Strategy, which aims to end the global TB epidemic by 2030 in line with the Sustainable Development Goals (SDGs) target, with a 90% reduction in TB deaths and an 80% reduction in TB incidence compared to 2015 levels. The End TB Strategy also has a high-level target of zero TB-affected families suffering from catastrophic costs due to the disease. This target is in line with the SDG target of universal health coverage (UHC), as well as the broader SDG agenda, which recognizes the intrinsic link between poverty and health. This target is being operationalized at country level with an increased focus on social protection and poverty alleviation, as well as recognition for the need for rigorous measurement of patient costs.

There has been previous work undertaken to measure patient costs in the field of TB, including in Myanmar [4,5,6,7,8,9,10]. However, a systematic review of TB patient costs found the majority of these studies were outdated, conducted in high income settings, suffered from small sample sizes, and had inconsistent methodology [11]. Furthermore, many of these studies did not assess costs alongside patient or household income, and therefore did not allow for a measure of the financial repercussions for TB-affected individuals.

A WHO Global TB Programme Task Force on TB Patient Cost Measurement agreed a standardized methodology for this work and produced a questionnaire and protocol to be adapted by countries. Myanmar was the first country to utilize this new methodology and tool. Myanmar is a lower-middle income country and one of the top 30 high TB burden countries, with 140,700 notified TB patients in 2015. This Myanmar patient costs survey utilized a large, nationally representative sample and included collection of disaggregated cost data and a set of measures of individual and household income. 

## 2. Methods

The survey design, objectives and methodology were adapted from the WHO protocol [12]. A nationally representative cluster-based sample survey was conducted in Myanmar in late 2015. With a design effect of 2.0, an estimated catastrophic costs prevalence of 30%, and a precision level of 4%, the required sample size was 1004. A total of 25 facilities were randomly selected by a probability proportional to size (PPS) method applying the TB case notification in 2014 for each facility (40 patients at each of 25 facilities) [13]. Within each selected facility, each patient who gave consent was interviewed about his or her TB-related spending, treatment history, household income, social consequences of the disease, and other key concerns. Patients were excluded if they were within the first two weeks of either their intensive or continuation phase of TB treatment, as it was considered they would not be able to provide enough cost information for the respective phase of care to obtain an accurate estimate of respective costs for that phase of care. 

### 2.1. Patient Costs

In this analysis, total costs include direct costs, both medical (e.g., drugs) and non-medical (e.g., transport), as well as indirect costs, which represent the value of a patient’s time spent seeking care. Direct medical costs are net of any reimbursements a patient received from health insurance. Direct non-medical costs are net of any benefit from the travel subsidy program in place, if reported by the patient. Less easily estimated are the indirect costs of TB disease. These are particularly important, as it has been shown that lost income exceeded direct medical costs by a factor of almost three in Uganda and South Africa [14,15]. Further, a systematic literature review estimates that 60% of the total cost incurred by a patient is the loss of income as a result of TB disease [2]. Indirect costs are defined as the difference in pre- and post-disease household annual income. As a sensitivity analysis, a second measure of indirect costs was undertaken using the human capital approach. Following this approach, an individual’s time is valued by multiplying the number of hours a patient spends seeking and receiving care by an hourly wage rate. Each survey respondent is assigned an hourly wage rate based on income/asset questions in the survey, as described below. Asset questions were validated using the Myanmar Household Income and Expenditure Survey (HIES). This human capital approach may be conservative as it focuses on the reported time spent seeking and receiving care but does not value the productivity lost due to the disease when not actively pursuing care. 

Additionally, many individuals seek care from traditional healers prior to arriving at a facility within the NTP network. A study of tuberculosis patients in Malawi found that almost 40% had done so before arriving at a government facility [16]. This indirect treatment pathway can lead to a significant cost incurrence before TB disease is correctly diagnosed. This was highlighted in work by Croft and Croft, in which data for Bangladeshi TB patient costs were analyzed, and it was found that they spent an average of USD 130 before reaching the TB clinic [17]. To put this in perspective, the GDP per capita of Bangladesh during the year of the study was USD 396. As a result, the questionnaire includes items pertaining to the entire diagnostic pathway.

### 2.2. Assessing Income

The primary measures are self-reported individual and household annual income. Income needed to be estimated for individuals who are not part of the formal economy and/or cannot accurately report annual wages. Therefore, in addition to asking for personal and household income, the questionnaire also inquired about household asset ownership and dwelling characteristics. We were able to estimate each household’s annual income using these items, utilizing the relationship between them and income for those who had complete data on these items. 

### 2.3. Extrapolation of Resource Utilization

The questionnaire was cross-sectional, with each patient being interviewed once after starting their TB treatment. To minimize recall bias, each patient was only asked about their current treatment phase, and about the costs incurred during their last visit. Per period costs were then calculated by multiplying the cost per visit against the number of visits reported during that period.

As a result, each patient’s costs and time spent for the entire TB episode were estimated using a combination of their reported costs and time and the responses of other patients. We hypothesized that patients’ resource utilization differed greatly in the two treatment phases: intensive and continuation. For this reason, within a treatment phase (either intensive or continuation) it was assumed that a patient’s costs were constant per day. For example, if a patient incurred USD 300 of costs after completing half of their intensive phase treatment, we assigned them a total intensive phase cost of USD 600. To estimate the direct costs for the treatment phase the patient was not currently in, we assigned them the median direct cost for respondents in that phase. Medians were calculated and assigned separately for drug-susceptible and MDR-TB patients due to their hypothesized differences in resource utilization. Median values of costs and time were seen as more conservative than means due to the skewed distributions of both costs and time spent seeking care.

### 2.4. Defining Catastrophic Costs

The definition of whether a household had incurred catastrophic costs was if the total costs were more than 20% of the total household income (pre-disease). This cut-off point is based on a study undertaken by Wingfield et al., where patients who had costs greater than 20% of their household income had inferior treatment outcomes [18]. Each TB-affected household was defined as either exceeding this threshold or not. In sensitivity analyses, this original threshold was varied to see how it affected the final percentage of households incurring TB-related catastrophic costs. 

### 2.5. Analyses

We report total costs by type (direct-medical, direct non-medical, and indirect) as well as with and without the cost of caregiver time included. By disaggregating total costs into its components, we hope to target programs or policies to the Myanmar context. For example, if most of the costs are being incurred before diagnosis, this would support an active case finding (ACF) program in which individuals with TB are actively sought out, usually among high-risk groups such as refugees, rather than waiting for them to seek care.

We also examine whether the TB-affected household had to undergo any kind of dissaving because of TB. This includes using savings, selling assets, or borrowing to cope with the costs of the disease. The term highlights a reduction in the financial strength of a household, in the same way that saving increases a household’s resilience to financial shocks. Previous work has shown an association between dissaving and costs, both total and relative to income, for TB patients in India [19].

Risk factors that were thought to be conceptually linked to incurring catastrophic costs (determined by (1) the previous smaller-scale studies [10], (2) the association identified by the univariate models, and (3) logical reasons according to the Myanmar culture) were tested using logistic regressions. These predictors included demographics, health-seeking behavior, household income, drug resistance status, insurance status, and reported social protection benefits. Costs were converted to United States Dollars (USD) using the average annual exchange rate during enrolment of USD 1 = 1282.65 Myanmar kyat (oanda.com). All analyses were conducted in Stata 13 (StataCorp LLC, College Station, TX, USA).

## 3. Results

Table 1 shows descriptive statistics for the study population of 967 eligible survey respondents. Sixty-six patients were being treated for MDR-TB at the time of the survey. On average, patients reported an annual household income of USD 2173 before TB treatment. The 288 new patients who were in the intensive phase of treatment reported on average a 6.85-week delay from onset of symptoms to diagnosis. However, this length of time varied greatly from 0 to 72 weeks.

Table 2 shows the model of care in Myanmar and highlights the average number of visits for survey respondents. The discrepancy of burden between DS-TB and MDR-TB patients can clearly be seen. MDR-TB patients have on average 447 visits during their TB treatment. Most of these visits are for DOT, with many patients reporting a daily visit for 20 months.

On average, patients incurred USD 759 as a result of their TB. These costs included USD 130 of direct medical expenditure, USD 265 of direct non-medical spending, and USD 365 of lost income due to the disease (Table 3). Of the direct non-medical spending, additional food and/or nutritional supplements comprised the largest portion of total spending. Spending on nutritional supplements and/or food outside the patient’s normal diet accounted, on average, for over one third of total spending for MDR-TB patients and approximately a quarter of total spending for drug-susceptible TB patients. 

**Figure 1:**
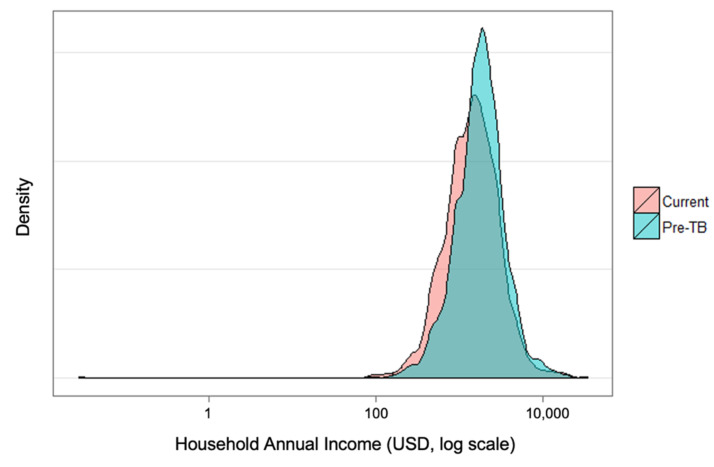
Distribution of annual household income pre-TB diagnosis and at time of survey, in 2015 USD.

Figure 1 shows the change in distribution of income of households before their TB diagnosis and at the time of the survey. There is a marked statistically significant shift in incomes, and this difference served as a proxy for indirect costs in the output approach. Under this approach, 60% (575/965) of TB-affected households experienced catastrophic costs due to TB at the 20% threshold. Under the human capital approach, 45% of TB-affected households (433/965) faced catastrophic costs. 

**Figure 2:**
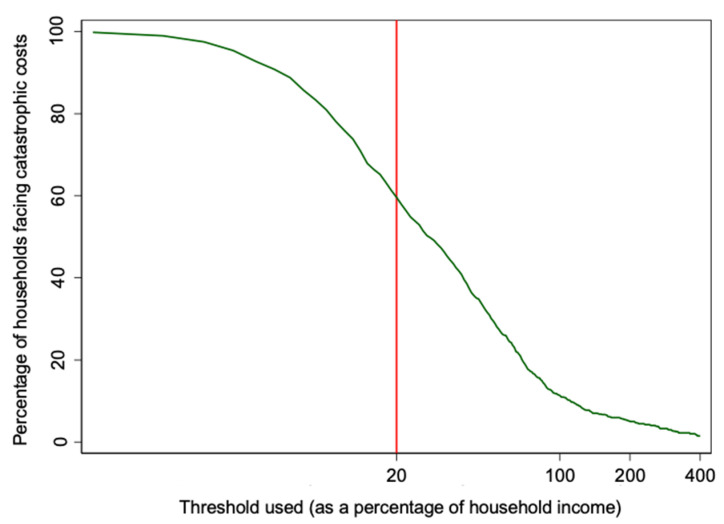
Sensitivity analysis of threshold used for determining catastrophic costs (output approach).

The median percentage of household income spent on TB was 27.8% with a mean of 57.4%. There was again a wide variation in this measure, with the percentage ranging from 1% of annual household income to over 18 times annual household income. Unsurprisingly, as the percentage of household income threshold is increased, the resulting percentage of households deemed to be facing catastrophic costs decreases (Figure 2). Even at a threshold of 100% of annual household income, 11.4% of households surveyed would be determined to be confronting catastrophic costs due to TB.

TB patients in Myanmar are also utilizing burdensome coping strategies, with approximately two-thirds using savings, borrowing, or selling assets to help pay for care (Table 4). However, only one quarter of the sample reported that TB had either a “serious” or “very serious” financial impact on the household.

Table 5 shows odds ratios for facing catastrophic costs (under both measurements of income loss) as well as use of at least one coping strategy resulting from univariate logistic regressions using patient characteristics. Measures with significant relationships to these outcomes are age, sex, and long diagnosis delay (output approach only), whether a patient is undergoing treatment for MDR-TB (human capital approach only), and the income quintile of the household (all models). Under the output approach, patients from the poorest quintile have 6.1 times the odds of those in the wealthiest quintile of experiencing catastrophic costs. We could not evaluate the effect of being treated for MDR-TB using logistic regression under the output approach because all MDR-TB patients experienced catastrophic costs under this approach. However, using the human capital approach MDR-TB patients were approximately 45 times more likely to experience catastrophic costs due to TB. 

## 4. Discussion

Despite policies of free public sector TB care in Myanmar, it is clear that many TB-affected households are experiencing catastrophic costs as a result. This effect can be seen particularly with patients who are male and elderly and is true regardless of which of the two methods of patient time valuation is used. Even under the most conservative approach, where indirect costs are not counted, over 35% of households are estimated to be facing catastrophic costs due to TB (not shown). People with TB/HIV face similar catastrophic costs as TB patients in this study. This can be explained by the fact that most public facilities provide both TB and HIV services, enabling patients with TB/HIV to receive similar care to those with TB only. In addition, there are strong TB/HIV collaborative activities including cross-referral in nearly all townships across country, coupled with a scaling up of ART decentralization services.

This study was limited to patients who were treated under the NTP network, which includes only a portion of the private sector. This excludes both TB-affected individuals who do not seek care altogether, as well as those who rely on private sector facilities not linked to the NTP. The reason for this limitation is that patients who are treated elsewhere are rarely notified and registered, and thus were not reachable for a survey. Despite higher costs, TB patients in Myanmar often utilize the private sector because of perceived higher quality care and/or shorter waiting times. Surprisingly, previous work has shown that in urban parts of Myanmar, TB patients who seek care in the private sector are poorer than those in the general population [20]. If this assumption holds true, the exclusion of private sector patients may lead to an underestimation of the percentage of patient households that experience catastrophic costs due to TB. Furthermore, patients were surveyed as they arrived at the health facility in each sampled township. This group of TB patients may be composed of those that are more frequent utilizers of the health system, thus generating higher costs. A patient receiving community DOTS was less likely to go to the health facility and thus less likely to be included in the survey.

A major issue with the estimation of total patient costs incurred is recall bias. We attempted to minimize this bias by only asking patients about their current treatment phase. However, scaling up the cost of a patient’s treatment phase depending on the amount of treatment remaining in that phase ignores how patients may adapt over time. Admittedly the extrapolation of costs is crude; however, it was not feasible to continuously follow patients to collect costs as they were incurred. 

The survey also fails to capture any form of payment that is not easily costed. It is often the case in Myanmar that patients who seek care in the private sector will pay for services with goods in-kind.

Moreover, the indirect costs of TB for the patient and the household can extend well beyond the treatment period, and for people who are declared cured of TB. People may be left with short- or long-term sequelae of the disease. Effects of coping mechanisms, such as selling household assets or taking children out of school, can impair household economy for years. For the documentation of long-term need of social and economic support for TB-affected households, measures of costs need to have a longer-term time-window than the present indicator.

Despite the limitations, this study was a nationally representative cluster-based sample survey, which was conducted as one of the first implementation experiences with the survey methodology based on WHO guidelines/references. To assess the validity of Myanmar patient cost survey results, we used sensitivity analysis of the threshold used for determining catastrophic costs and significantly improved survey methodology. Myanmar was the first country globally to successfully carry out the survey, followed by Vietnam. Since then, other countries have had opportunities to learn from Myanmar’s experiences, and a handbook on how to implement these types of studies has been published by WHO [21].

A multi-sectoral workshop was held in Naypyidaw to discuss the findings of this survey and consider how various governmental departments could mitigate patient costs. There are several current measures in place to lessen the cost burden on patients. The current move towards UHC, including implementation of the essential health package, may improve access to healthcare and reduce costs of consultations and non-TB drugs. Furthermore, there is a national policy for free TB diagnostic tests and drug treatment, including first-line, second-line, and ancillary drugs to manage side effects. However, as direct medical expenditure accounted for only 17% of total costs, it is unlikely that universal health coverage and/or free drugs will reduce costs to levels that do not place an unreasonably heavy burden on TB-affected households. 

To combat travel costs, decentralization of care for both drug-sensitive and drug-resistant patients continues to progress. There are also nutritional support and travel subsidy programs that are in place for MDR-TB patients. High uptake of these programs was confirmed by the survey, with 60 of 66 MDR-TB patients reporting participation in at least one support program. At the dissemination meeting, the Ministry of Social Welfare agreed to include MDR-TB patients in their social protection scheme by 2020. Furthermore, the Global Fund approved an additional USD 15 per month for all MDR-TB patients. The Ministry of Social Welfare Relief and Resettlement has approved support for MDR-TB patients in terms of transportation cost beyond 2023 (currently covered by GF until 2023) in their National Social Protection Plan. These improvements in social support are critical, given the large share of total costs that were due to a patient’s lost income. 

In short, this study clearly shows that many TB-affected households are experiencing catastrophic costs, and more TB-specific social protection programs in patient-centered healthcare are needed in Myanmar.

## Figures and Tables

**Table 1:** Descriptive statistics of survey sample by MDR status and overall.

	MDR-TB (*n* = 66)	Drug-Susceptible TB (*n* = 901)	All (*n* = 967)
**Sex**			
Male	40 (60.6%)	555 (61.9%)	595 (61.8%)
Female	26 (39.4%)	342 (38.1%)	368 (38.2%)
Unknown	0 (0%)	4 (0.4%)	4 (0.4%)
Age in years (SD)	36.1 (13.3)	34.7 (20.8)	34.8 (20.3)
Health insurance	0 (0%)	4 (0.4%)	4 (0.4%)
**Phase**			
Intensive	30 (45.5%)	348 (38.6%)	378 (39.1%)
Continuation	36 (54.5%)	553 (61.4%)	589 (60.9%)
**HIV Status**			
Positive	6 (9.1%)	53 (5.9%)	59 (6.10%)
Negative	41 (62.1%)	674 (74.8%)	715 (73.9%)
Unknown	19 (28.8%)	174 (19.3%)	193 (20.0%)
**Retreatment status**			
New	14 (21.2%)	790 (87.7%)	804 (83.1%)
Retreatment/Relapse	52 (78.8%)	111 (12.3%)	163 (16.9%)
Monthly household income in USD (SD)	212.8 (163.8)	179.8 (150.3)	182.0 (151.4)
Diagnosis delay in weeks (SD) *	6.6 (6.2)	6.9 (8.9)	6.85 (8.9)

* Only assessed for 288 new patients in intensive phase.

**Table 2:** Model of care for survey sample.

	MDR-TB	DS-TB	All
	(*n* = 66)	(*n* = 901)	(*n* = 967)
Hospitalized at time of interview, N (%)	0 (0)	11 (1.2)	11 (1.1)
Hospitalized during current phase, N (%)	12 (18.2)	111 (12.3)	123 (12.7)
Days hospitalized during current phase, Mean (SD)	47.1 (98.0)	9.6 (15.5)	13.0 (33.6)
Hospitalized in previous episode(s), N (%)	4 (6.1)	10 (1.1)	14 (1.5)
Days hospitalized in previous episode(s), Mean (SD)	25.3 (24.2)	22.9 (44.9)	23.6 (39.1)
Number of visits per episode: Total, Mean (SD)	447.4 (147.5)	59.4 (69.2)	85.9 (124.5)
Number of visits: DOT, Mean (SD)	397.2 (127.9)	48.2 (69.1)	72.2 (115.5)
Number of visits: follow-up, Mean (SD)	24.1 (27.8)	3.5 (5.0)	5.0 (10.2)
Number of visits: drug pick-up, Mean (SD)	26.2 (81.4)	7.3 (5.1)	8.6 (22.2)
Number of visits pre-diagnosis, Mean (SD)	0.6 (1.4)	2.2 (2.2)	2.0 (2.1)
Treatment duration months Mean (SD)	20.2 (0.9)	6.5 (1.3)	7.5 (3.7)

**Table 3:** Summary of detailed costs in 2015 USD.

	MDR-TB (*n* = 66)	Drug-Susceptible-TB (*n* = 901)	Total (*n* = 967)
	Median (Min–Max)	Median (Min–Max)	Median (Min–Max)
**Before Diagnosis**			
Medical	21.4 (5.1–85.8)	12.2 (0–818.6)	12.2 (0–818.6)
Non-medical	5 (1.01–35.0)	5 (0–261.9)	5 (0–261.9)
**After Diagnosis**			
Medical	220.8 (51.98–3706.3)	80.8 (19–1219)	80.8 (19–3706.3)
Travel	131.7 (-339.1–2296.5)	28.8 (11.7–4081.7)	29.5 (-339.1–4081.7)
Accommodation	27.3 (9.5–235.2)	1.7 (1.68–194.3)	1.7 (1.68–235.2)
Food	695 (183.9–3611.6)	102.6 (17.6–4137.1)	112 (17.6–4137.1)
Patient and caregivers’ time	561.3 (0–4677.8)	0 (0–93.5)	0 (0–224.1)
**Total**	1157.52 (46.63–4927.0)	223.12 (46.63–5168.5)	238.12 (46.63–5168.5)

**Table 4:** Coping mechanisms and social consequences.

	Income Quintiles					
	Poorest (*n* = 199)	Less Poor (*n* = 201)	Average (*n* = 181)	Less Wealthy (*n* = 222)	Wealthiest (*n* = 163)	Overall (*n* = 967)
**Coping Strategies**						
Loan	84 (42.2%)	84 (41.8%)	55 (30.4%)	61 (27.5%)	30 (18.4%)	314 (32.5%)
Dissaving	68 (34.2%)	72 (35.8%)	68 (37.6%)	85 (38.3%)	63 (38.7%)	356 (36.8%)
Sale of Assets	60 (30.2%)	63 (31.3%)	39 (21.6%)	42 (18.9%)	25 (15.3%)	230 (23.8%)
Any of the three above	149 (74.9%)	141 (70.2%)	114 (63.0%)	127 (57.1%)	91 (55.8%)	623 (64.4%)
Food insecurity	6 (3.0%)	5 (2.5%)	3 (1.7%)	1 (0.5%)	0 (0.0%)	15 (1.6%)
Divorce or separation from spouse/partner	2 (1.0%)	0 (0.0%)	1 (0.6%)	2 (0.9%)	0 (0.0%)	5 (0.5%)
Loss of Job	3 (1.5%)	3 (1.5%)	2 (1.1%)	6 (2.7%)	4 (2.5%)	18 (1.9%)
Child interrupted schooling	12 (6.0%)	4 (2.0%)	8 (4.4%)	3 (1.4%)	6 (3.7%)	33 (3.4%)
Social exclusion	12 (6.0%)	13 (6.5%)	6 (3.3%)	11 (5.0%)	5 (3.1%)	47 (4.9%)
Any days of work lost	99 (49.8%)	119 (59.2%)	114 (63.0%)	124 (55.9%)	94 (57.8%)	551 (57.0%)
**How big a financial impact did TB have on your household?**						
No impact	40 (20.1%)	27 (13.4%)	37 (20.4%)	52 (23.4%)	49 (30.1%)	205 (21.2%)
Little impact	49 (24.6%)	65 (32.3%)	56 (30.9%)	67 (30.2%)	42 (25.8%)	279 (28.9%)
Moderate impact	49 (24.6%)	57 (28.4%)	43 (23.8%)	49 (22.1%)	39 (23.9%)	238 (24.6%)
Serious impact	47 (23.6%)	45 (22.4%)	35 (19.3%)	39 (17.6%)	25 (15.3%)	191 (19.8%)
Very serious impact	14 (7.0%)	6 (3.0%)	10 (5.6%)	15 (6.8%)	7 (4.3%)	52 (5.4%)

**Table 5:** Odds ratios of experiencing catastrophic costs under the two indirect cost methods and for engaging in any coping strategies ^a,b^.

	Catastrophic Cost Incurred (Output Approach)	Catastrophic Cost Incurred (Human Capital Approach)	Any of the Three Coping Strategies
Age	1.01 *** (1.00, 1.02)	1.00 (1.00, 1.01)	1.00 (1.00, 1.01)
Sex			
Male	1.34 * (1.03, 1.75)	1.06 (0.82, 1.38)	1.06 (0.81, 1.39)
Female	Reference	Reference	Reference
MDR-TB	N/A	45.1 *** (11.0, 185.6)	1.51 (0.86, 2.64)
Long delay (>4 weeks before diagnosis) ^c^	1.67 * (1.02, 2.75)	1.16 (0.72, 1.86)	1.61 (0.98, 2.64)
HIV	1.49 (0.84, 2.65)	1.04 (0.61, 1.77)	1.18 (0.66, 2.10)
Income Quintile			
Poorest	6.14 *** (3.74, 10.07)	19.82 *** (11.60, 33.85)	2.36 *** (1.51, 3.68)
Less Poor	1.87 ** (1.23, 2.85)	4.60 *** (2.89, 7.33)	1.86 ** (1.21, 2.86)
Average	1.09 (0.71, 1.67)	2.22 *** (1.37, 3.59)	1.36 (0.87, 2.07)
Less Wealthy	1.09 (0.73, 1.63)	1.06 (0.65, 1.73)	1.06 (0.70, 1.59)
Wealthiest (Reference)	Reference	Reference	Reference

^a.^ * < 0.05, ** < 0.01, *** < 0.001. ^b.^ All results are from univariate models ^c.^ Only assessed for 288 new patients in intensive phase.

## Data Availability

The data supporting the findings of this study are available within the article.
